# African Crop Calendar: A geo-referenced dataset of crop growing seasons across diverse agro-ecological regions

**DOI:** 10.1038/s41597-026-07906-9

**Published:** 2026-08-25

**Authors:** Katharina Waha, Sophie Rötzer, Koen De Vos, Uwe Grewer, Andrew Tomes, Altaaf Mechiche-Alami

**Affiliations:** 1 Centre for Climate Resilience, University of Augsburg, Augsburg, Germany; 2 Department of Earth and Environmental Sciences, KU Leuven, Leuven, Belgium; 3 Biodiversity and Natural Resources Program, International Institute for Applied Systems Analysis (IIASA), Laxenburg, Austria; 4 Environmental Intelligence, Vlaamse Instelling voor Technologisch Onderzoek (VITO), Mol, Belgium; 5 Centre for Sustainable Agricultural Systems, University of Southern Queensland, Toowoomba, Australia; 6 Evans School Policy Analysis & Research (EPAR), Daniel J Evans School of Public Policy, University of Washington, Seattle, USA; 7 Lund University Centre for Sustainability Studies (LUCSUS), Lund University, Lund, Sweden

**Keywords:** Plant physiology, Agriculture, Geography

## Abstract

Large-scale agricultural household surveys provide essential data on farming households’ socioeconomics, land management, and agricultural indicators, and allow detailed micro-scale comparisons while ensuring national representativeness. This study introduces cleaned, harmonised, and validated survey data describing the growing season of several hundred crops from six African countries between 2009–2019. It complements existing datasets by including geographical identifiers, facilitating the linkage with climate, soil, or Earth observation data. Collected through the LSMS-ISA program, the surveys cover crop planting and harvesting dates, anonymized field locations, and crop species grown. The data were harmonised by assigning consistent variable names, correcting inconsistencies, standardising crop names, and creating unique plot identifiers. This comprehensive dataset offers novel insights into African cropping systems and growing seasons and provides a basis for assessing drivers of shifts in growing seasons.

## Background & Summary

Agricultural household surveys provide direct insights into farming households’ socioeconomics, the farm structure, and land management strategies in addition to key agricultural development indicators such as crop yield and market participation. Household surveys bring several advantages over national production statistics, such as observations on the individual and plot-level that facilitate inferences and comparisons at the scale where decisions are made. Other benefits are internal consistency as multiple agricultural, socio-economic and demographic indicators can be derived from the same sample, and representativeness if collected with a nationally representative sampling framework.

Here we cleaned, harmonised, standardised and validated unique longitudinal household survey data describing the planting time, harvest time and length of cropping period for several hundred crops in six African countries over the 10-year time period 2009 to 2019^1^. Our focus on cropping period is important as this is an essential characteristic of cropping systems influencing crop development and production. Existing cropping period or crop calendar data are either aggregated across several land cover and crop classes^2,3^, limited to few crops^4,5^, based on expert knowledge such as the FAO’s Global Information and Early Warning System on Food and Agriculture (GIEWS) country briefs and Crop Calendar platform and the USDA’s crop calendar charts, or simulated based on climatic constraints^6–9^. To our knowledge, there is no harmonized longitudinal, crop-specific, field-level data set on planting and harvesting times.

Our efforts complement the work of other research teams producing survey-based datasets such as RHoMIS^10,11^, which includes multiple agricultural, welfare, productivity and economic indicators for selected sites across the world with data collection still ongoing but not designed to be representative on the national scale. Other similar datasets are the CEEPA survey data^12^ collected 20 years ago, Grow-Africa^13^ which includes georeferenced observations of crop yields across Africa and AgDevIndicators^14^, which includes multiple agricultural indicators for selected African countries but was not designed to consider crop planting and harvesting times.

The dataset^1^ is spatially explicit, including geographical identifiers at different levels of aggregation, to link to other spatial datasets such as climate, soil, and management data, Earth Observation data, sub-national to national statistics or land use and land cover classifications. Our motivation for creating this dataset is to improve the integration of this largely untapped data source into interdisciplinary research by cleaning, processing and sharing data to accelerate the pace of scientific discovery. We provide a data description and a basic narrative on temporal and spatial trends in cropping systems and growing seasons in a subset of African countries.

Highlights:

- We present a new crop calendar dataset, created by harmonizing agricultural information from previously underused nationally representative household surveys.
- This dataset has broad spatial (six African countries) and temporal coverage (2009–2019) and higher spatial granularity than existing crop calendars.
- The dataset is designed to identify and monitor spatial and temporal trends in growing seasons for a multitude of annual, perennial, tree, and fodder crops.
- The dataset can be integrated with studies on agriculture, land use, climate, phenology, and rural households’ land management.

## Methods

### Original datasets

The agricultural household surveys are part of the Living Standards Measurement Study - Integrated Surveys on Agriculture (LSMS-ISA) programme^15^. They are longitudinal and cross-sectional surveys conducted between 2010 and 2020, including repeated observations of the same households and a lot of data points from many different individuals of the same population. Technical reports on the sampling strategies, survey design, fieldwork and data entry and the original datasets can be found in the World Bank Microdata Catalogue by searching for the dataset reference ID given in the metadata file or the DOI from Table 1. The panel nature of the LSMS-ISA surveys provides opportunities for long-term and comparable production of indicators and statistics over time. An initial scoping analysis showed that 19 of the 30 LSMS-ISA survey waves include information on both crop planting and crop harvest dates, representing multiple years between 2009 and 2019.

**Table 1:** Reference ID and DOI of the available national survey datasets.

Dataset ID	Country	Survey Year	Planting Dates	Harvest Dates	DOI of the dataset
*Included*					
ETH_2011_ERSS_v02_M^15^	Ethiopia	2011	TRUE	TRUE	10.48529/80xt-9m68
ETH_2013_ESS_v03_M^17^	Ethiopia	2013	TRUE	TRUE	10.48529/mccp-y123
ETH_2015_ESS_v03_M^18^	Ethiopia	2015	TRUE	TRUE	10.48529/ampf-7988
ETH_2018_ESS_v03_M^19^	Ethiopia	2018	TRUE	TRUE	10.48529/k739-c548
MWI_2010_IHS-III_v01_M^20^	Malawi	2010	TRUE	TRUE	10.48529/w1jq-qh85
MWI_2010-2013_IHPS_v01_M^21^	Malawi	2013	TRUE	TRUE	10.48529/4q9f-2288
MWI_2016_IHS-IV_v04_M^20^	Malawi	2016	TRUE	TRUE	10.48529/g2p9-9r19
MWI_2019_IHS-V_v05_M^22^	Malawi	2019	TRUE	TRUE	10.48529/yqn3-zv74
MLI_2014_EACI_v03_M^23^	Mali	2014	TRUE	TRUE	10.48529/qqam-mn86
MLI_2017_EAC-I_v03_M^24^	Mali	2017	TRUE	TRUE	10.48529/0v50-h966
NER_2014_ECVMA-II_v02_M^25^	Niger	2014	TRUE	TRUE	10.48529/3xnb-sd96
NGA_2015_GHSP-W3_v02_M^26^	Nigeria	2015	TRUE	TRUE	10.48529/7xmj-q133
NGA_2018_GHSP-W4_v03_M^27^	Nigeria	2018	TRUE	TRUE	10.48529/1hgw-dq47
NGA_2023_GHSP-W5_v01_M^28^	Nigeria	2023	TRUE	TRUE	10.48529/zd5s-tj25
UGA_2011_UNPS_v02_M^29^	Uganda	2011	TRUE	TRUE	10.48529/wjg5-bg56
UGA_2013_UNPS_v02_M^30^	Uganda	2013	TRUE	TRUE	10.48529/c1c4-h654
UGA_2015_UNPS_v02_M^31^	Uganda	2015	TRUE	TRUE	10.48529/hm2z-2k12
UGA_2018_UNPS_v02_M^32^	Uganda	2018	TRUE	TRUE	10.48529/ttyg-m774
UGA_2019_UNPS_v03_M^33^	Uganda	2019	TRUE	TRUE	10.48529/nqzx-f196
*Not Included*					
BFA_2014_EMC_v01_M	Burkina Faso	2014	FALSE	FALSE	10.48529/E39G-M952
NER_2011_ECVMA_V01_M	Niger	2011	TRUE	FALSE	10.48529/bp16-s524
NGA_2010_GHSP-W1_v03_M	Nigeria	2010	TRUE	FALSE	10.48529/y9e2-b753
NGA_2012_GHSP-W2_v02_M	Nigeria	2012	TRUE	FALSE	10.48529/kxpy-aa72
TZA_2008_NPS-R1_v03_M	Tanzania	2008	FALSE	TRUE	10.48529/hz8s-3489
TZA_2010_NPS-R2_v03_M	Tanzania	2010	FALSE	TRUE	10.48529/jm20-c742
TZA_2012_NPS-R3_v01_M	Tanzania	2012	FALSE	TRUE	10.48529/7vqv-5f71
TZA_2014_NPS-R4_v03_M	Tanzania	2014	FALSE	TRUE	10.48529/y3qj-d018
TZA_2019_NPS-SDD_v06_M	Tanzania	2019	FALSE	TRUE	10.48529/0y7d-1v78
UGA_2005-2009_UNPS_v03_M	Uganda	2005 and 2009	FALSE	FALSE	10.48529/9vep-e461
UGA_2010_UNPS_v03_M	Uganda	2010	FALSE	TRUE	10.48529/w2d8-t244

The datasets considered in the study are those with both planting and harvest dates available. From the Malawian panel dataset, MWI_2010-2013_IHPS_v01_M, only the 2013 data were used in this study, as the 2010 data were taken from the larger cross-sectional survey, MWI_2010_IHS-III_v01_M. We used data from the Ugandan dataset ´UGA_2011_UNPS_v02_M´0 (version 01) which has been updated to v02 in August 2025; the only difference is in one section of the dataset that was not used here.

### Questionnaires and timing of field work

The surveys are typically organised into a household, community, agricultural and other sections with varying content by country and survey wave, although questionnaires from one wave were often used again in the next survey waves. Respondents in households were visited twice during the survey period and asked different questions depending on the main agricultural and economic activities. The purpose of the two-visit strategy is to minimise recall error related to conditions experienced during the agricultural season, accounting for plot input and labour use, to reduce the total length of the visit and to have adequate time to reach households that live in very remote areas. The data collected during the first visit typically refers to the time after the planting season (“post-planting questionnaire”) and the second visit is intended to collect data after the harvest season (“post-harvest questionnaire”). While planting dates are generally reported for a specific month, harvests can span longer periods, as some fields can have multiple harvests particularly for perennial, tree and fodder crops. As such, we report harvests as harvest seasons with a beginning and end month instead of a single event. As there is considerable variation in the onset and length of the main rainy seasons there are small variations in the timing of the field work between the survey years.

**Fig. 1:**
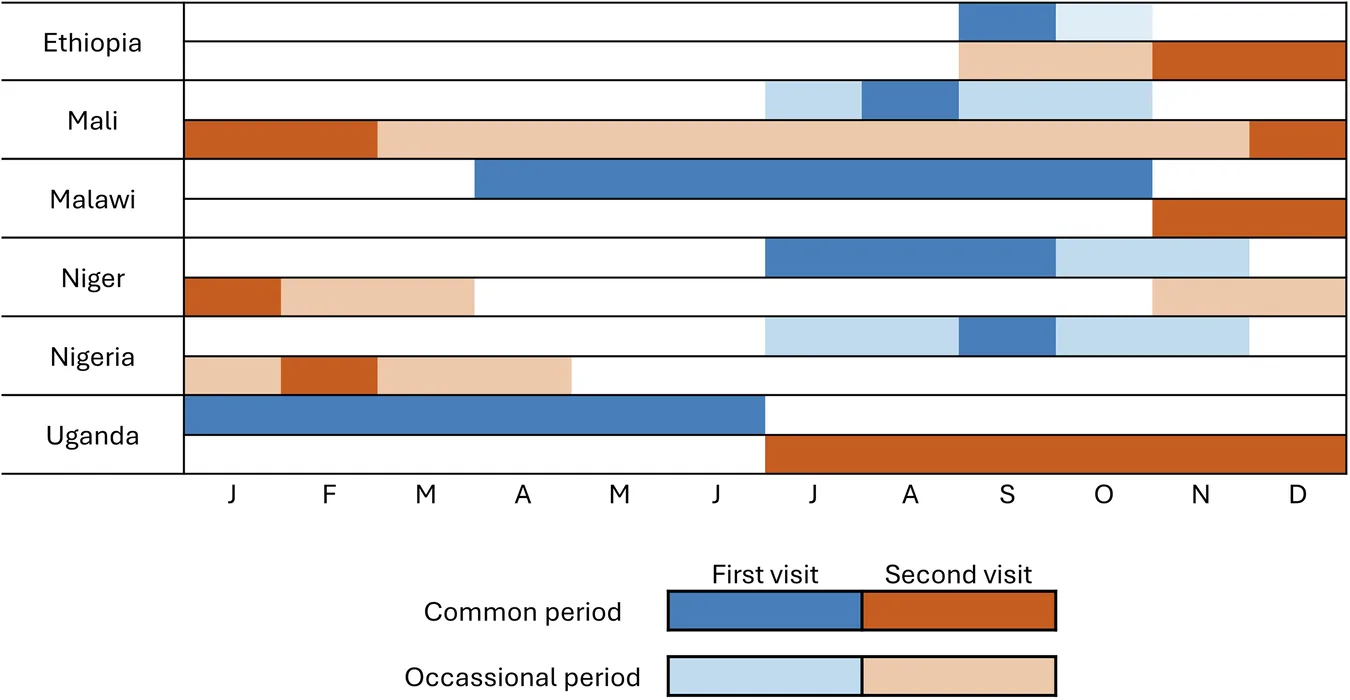
Beginning and end of typical survey cycles and timing of field work, by country and as indicated in the study description on the World Bank’s Microdata Library. The “common period” denotes the months consistently used for field work across different survey years, while the “occasional period” refers to additional months in some years extending beyond the typical field work window.

In Ethiopia, the two visits were conducted between September-October, sometimes through to December or March the next year and in January-February sometimes through to March-April (Fig. 1). In Uganda data collected during the two visits refers to the two main cropping seasons in January-June and July-December. In Malawi, the first visit was conducted during the post-planting period before the main November-April rainy season and the second visit approximately three months after the first visit in the post-harvest period, except for the Fourth and Fifth Integrated Household Surveys, when households were only visited once. In Mali and Nigeria, the two visits took place between July and October (sometimes through to November) and between December and February the following year (sometimes running through to April) to match the post-planting and post-harvest seasons with respect to the main rainy season. For every survey, we have extracted information from the household and agricultural sections, in particular about the identification of households and land units, the parcel or garden roster for main land units, the field, plot or parcel roster for information about sub-units of land and the timing of cultivation of crops.

### Data extraction and harmonisation

We generated the dataset^1^ through a stepwise data extraction, cleaning and harmonisation process guided by a written set of instructions and standardised templates for datasets and metadata generation. We first defined a target dataset format that clearly defined all focus variables and variable units. This template ensured the conversion of the diverging naming conventions across surveys to a harmonised set of variable names. While generally all target variables were recorded in the various input datasets, the specific definition of selected variables differs among surveys and survey waves. For example, some surveys exclusively record the main month in which harvesting took place, while other surveys gather the first month of the harvest and the last month of the harvest separately. The dataset template actively accommodates these differences in data structure and avoids the need to simplify data or reduce the level of detail as part of data extraction. We generate metadata of standardised format for each survey that transparently documents the full list of original variable identifiers in the source data, the original survey questions, and any potential transformation (e.g., in variable units) as part of the data extraction process.

In order to ensure consistency in individual researcher’s data extraction/harmonization efforts, we first created a smaller coordinating team that proposed and enforced the steps to take and provided consistent advice in unclear cases. Secondly, we implemented an internal review process where each survey’s extracted data was assigned to another researcher for review. Any upcoming issues were reported in separate issues in the jointly used GitHub repository and assigned to the responsible researcher. All authors conducted the stepwise data extraction and harmonisation process for a designated number of surveys. Each survey was assigned to a single researcher typically batched by country to leverage similarities between the surveys. As part of the data extraction process, we remained as close as possible to the source data. Specifically, in the first step, raw data were also kept when logically implausible (e.g. planting month = 26). Missing values were recoded to a unique identifier (“NA”) across all survey waves. While this might lead to a high prevalence of NA in the dataset, as data entries represent individual plots or land units, they still provide relevant information for the growing season of a household.

In a second step, we cleaned and further harmonised the full dataset containing all individual survey waves. The data cleaning aimed to retain all plausible values, even if unlikely, while excluding those that were unequivocally incorrect or logically impossible. Where records are duplicates, the record with the least information is deleted, and, if they are identical, then one of the records is arbitrarily retained. If a farmer plants and harvests the same crop twice but on two separate plots these are not duplicates and are not removed as identical as the two plots have two different plot identifiers and might be managed in a different way. Redundant records that only include household and spatial identifiers were removed. Item non-responses can occur when a respondent fails to provide data for one or more items in the questionnaire, either because of inability or refusal to answer, or irrelevant questions.

### Crop names and classifications

**Fig. 2:**
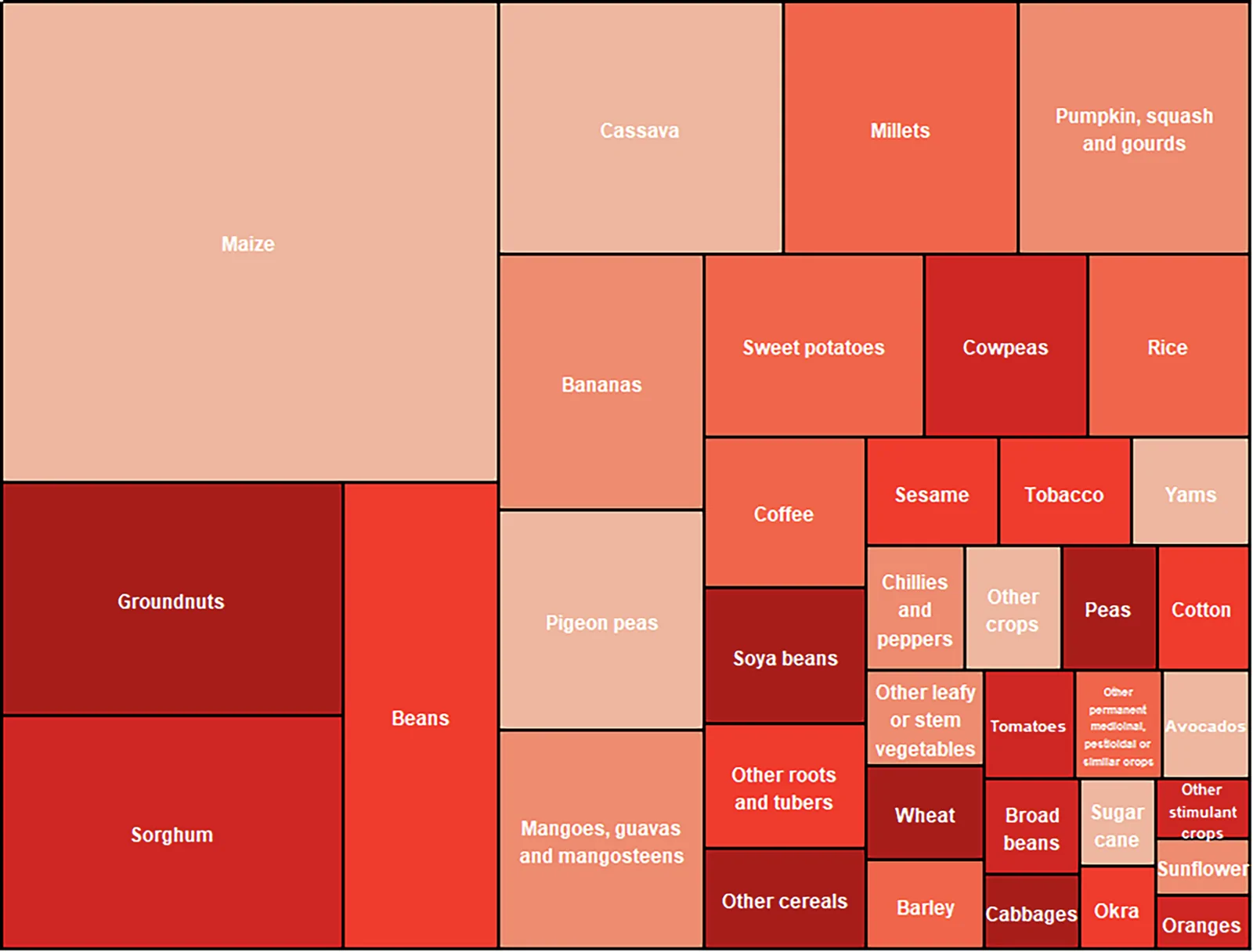
Most prevalent crops and crop groups with information on their planting and harvest dates in the dataset. Each nested rectangle represents the number of households cultivating at least one of their plots with the respective crop. There are over 350 individual crops in the dataset. The figure shows the crops and crop groups cultivated by at least 1,000 households.

The crop names are corrected for spelling mistakes and variations, with variants combined under the singular (e.g. “pinapples”, “pineapples” and “pineapple” have all been combined under “pineapple”). Further descriptions, variety names or other names of the crops have been placed in brackets (e.g. “pawpaw” and “papaya” are recoded as papaya (pawpaw). The final list of crops includes names of over 350 annual, perennial, tree, and fodder crops and, in some cases, names of local crop varieties. A lot of crops are rare or are given with their local names. We further generated a simplified crop name variable that exclusively identifies known crop types without any further attributes (e.g., “yam, white” and “yam, yellow” are recorded as “yam”) and assigned the value “other” to responses that could not be reliably identified as a known crop type. The latter facilitates aggregation by major crop types (156 simplified names), while abstracting from further detail. The most prevalent crops in the dataset are *maize*, *groundnuts*, *sorghum*, *beans*, and *cassava* (Fig. 2). The most prevalent vegetables excluding beans, peas, potatoes and yams are *pumpkins*, *squash and gourds*, *chillies and peppers*, *leafy and stem vegetables, tomatoes*, and *cabbages*. Other crops relevant for agricultural export and included in the dataset are *cocoa, cashew nuts, tobacco, coffee, cotton, tea*, and *oranges*. Other crops of national significance and included are *sorrel* in Niger, *enset* and *teff* in Ethiopia, and *mango* in Malawi. An additional file includes the lookup table highlighting the dataset’s crop names correspondence to those used by the FAO’s Indicative Crop Classification (ICC 1.1;^16^, pages 165–169).

### Geographic identifiers and geographic coordinates

**Fig. 3:**
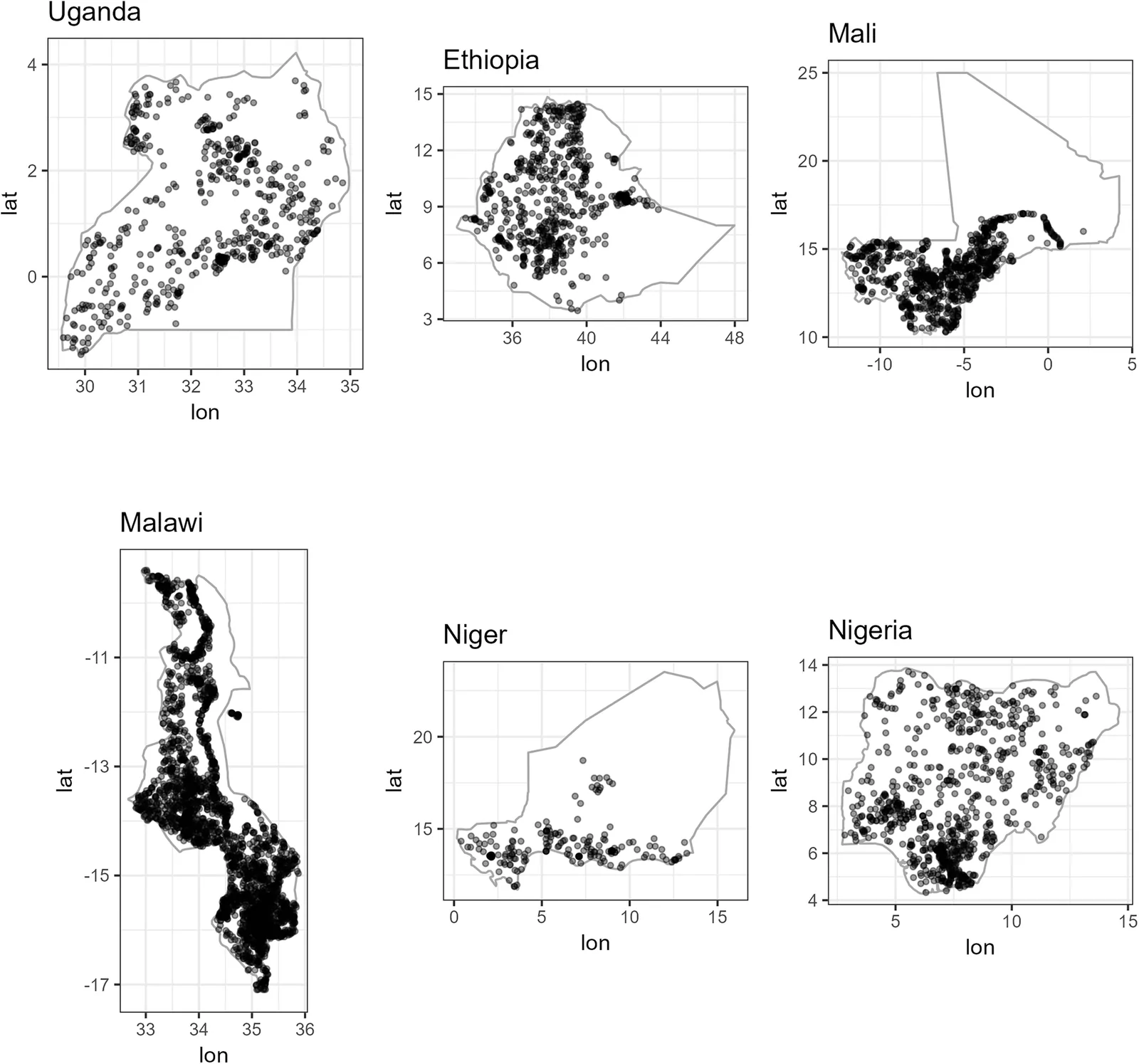
Location of unique geographic coordinates in all six countries included in the dataset. Geographic coordinates typically refer to the enumeration area. In addition to geographic coordinates, the dataset provides the names of administrative units as additional geographic identifier.

The original surveys reported geographic coordinates in decimal degrees. They mostly describe a centroid point for each enumeration area in Ethiopia, Mali, Niger, Malawi and Uganda (Fig. 3). This spatial data was collected and anonymized by the national statistics offices implementing each survey. The centroid point is calculated as the average of surveyed household GPS coordinates in each enumeration area. To preserve the confidentiality of households and communities, the national statistics agencies have applied a random offset within a specified range to the average enumeration area coordinates according to the methodology used in national demographic and health surveys, with some modifications. The coordinates were randomly fuzzed by up to 10 km in rural areas and 5 km in urban areas.

Enumeration areas are typically smaller than 2nd or 3rd-level administrative units or created from multiple administrative units. Uganda is a special case with geographic coordinates only available for the National Panel Survey 2011/12, but not for the later surveys. In Nigeria, geographic coordinates describe the centroid point of administrative level 4, however an unknown coding system is used for this administrative level. We use a new variable, GPS levels, to distinguish between the different spatial scale of geographic coordinates used in the different countries: GPS level 2 for households, GPS level 3 for enumeration area or community level or GPS level 4 if the spatial scale is unknown. Longitude and latitude were replaced by NA if both values were 0.

Other geographic identifiers are the names of the administrative units the households belong to. In Uganda, the administrative levels 1 to 4 are provided. In the other countries at least administrative levels 1 and 2 are given. They refer to different naming conventions for distinct geographic areas that form administrative subdivisions of a country (Table 2).

**Table 2:** Overview of the information in the dataset^1^ including administrative units (adm level) and number of households, crops and plots by country and year.

Country	Year	Adm level 1	Adm level 2	Adm level 3	Adm level 4	Households	Crops	Sample	Planting month	Start of harvest	End of harvest
Ethiopia	2011	10	55	178	0	2,828	70	29,303	84%	11%	10%
Ethiopia	2013	10	55	186	0	3,009	60	30,415	73%	87%	87%
Ethiopia	2015	10	55	185	0	2,901	61	30,341	53%	85%	85%
Ethiopia	2018	10	46	61	0	2,062	62	17,013	66%	60%	60%
**Ethiopia**		**10**	**55**	**191**	**0**	**8,021**	**70**	**107,072**	**69%**	**61%**	**61%**
Mali	2014	8	44	247	0	2,265	29	10,052	100%	95%	93%
Mali	2017	8	47	371	0	6,230	31	24,926	100%	100%	95%
**Mali**		**8**	**47**	**524**	**0**	**6,598**	**35**	**34,978**	**100%**	**99%**	**95%**
Malawi	2010	3	31	0	0	10,274	35	35,518	83%	97%	98%
Malawi	2013	3	31	0	0	2,619	33	13,899	80%	88%	88%
Malawi	2016	3	32	0	0	9,886	56	31,450	87%	98%	99%
Malawi	2019	3	32	0	0	9,241	73	43,589	82%	96%	97%
**Malawi**		**3**	**32**	**0**	**0**	**29,591**	**79**	**124,457**	**83%**	**96%**	**97%**
**Niger**	**2014**	**8**	**35**	**18**	**0**	**2,105**	**35**	**11,944**	**100%**	**77%**	**77%**
Nigeria	2015	6	37	350	0	2,867	47	11,810	91%	84%	77%
Nigeria	2018	6	37	349	0	3,446	42	15,270	94%	73%	66%
Nigeria	2023	6	36	374	0	3,322	48	10,553	95%	82%	70%
**Nigeria**		**6**	**37**	**570**	**0**	**5,837**	51	**37,633**	**93%**	**78%**	**70%**
Uganda	2011	103	172	367	500	2,384	34	57,272	98%	20%	20%
Uganda	2013	4	101	388	516	2,437	40	21,379	90%	73%	73%
Uganda	2015	102	439	629	886	2,607	39	22,782	28%	22%	24%
Uganda	2018	116	428	605	797	2,584	40	15,758	87%	78%	78%
Uganda	2019	120	436	633	0	2,473	38	16,403	90%	72%	72%
**Uganda**		**135**	**794**	**1354**	**1929**	**12,485**	**42**	**133,594**	**82%**	**42%**	**42%**
**Total**		**169**	**1004**	**2652**	**1929**	**61,827**	**197**	**462,347**	**82%**	**68%**	**67%**

We also present the share of available planting dates and harvest periods. The basic information document for each survey provides information on the sampling design, representativeness, percentage of administrative units sampled and selection of households in each administrative unit. They can be found in the World Bank Microdata Catalogue, when searching for the DOI or Reference ID of each survey.

### Consistent plot identifiers

Descriptions of agricultural fields vary between countries and often two to three different identifiers are used. This is because agricultural fields can be subdivided into smaller units of land for different uses or for use in different agricultural seasons or managed differently. These sub-divisions are referred to as “plots” in Malawi, Uganda and Nigeria, but as “fields” in Ethiopia or as “parcels” in Mali and Niger. Larger collections of plots may be referred to as “parcels” in Ethiopia and Uganda, or as “gardens” or “munda/minda” in Malawi. A garden in Malawi is a continuous piece of land not subdivided by a river or a path wide enough for an oxcart or vehicle.

**Fig. 4:**
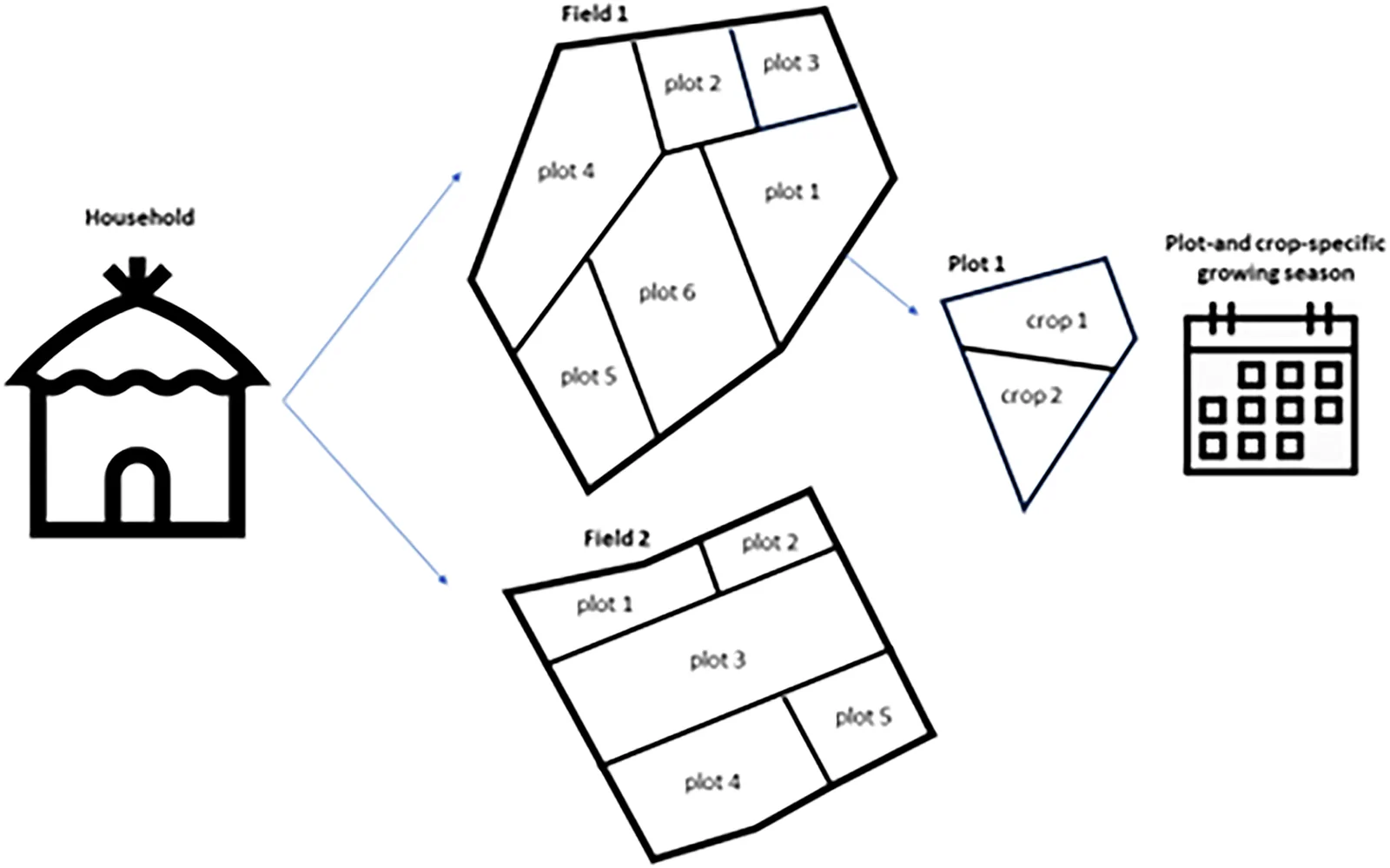
Three levels of spatial identifiers used in the harmonised dataset, from left to right household identifier, field identifier and plot identifier. Multiple crops may be cultivated on individual plots.

We created a standard format for identifying agricultural fields and their subdivisions in the harmonized dataset (Fig. 4). *Plots* are sub-divisions of *Fields* and are identified by the identifier of the household they belong to, and the *Field* they belong to. This means that field identifiers are used to identify a collection of *Plots* that share a set of characteristics and plot identifiers are used as the lowest level spatial identifier in the dataset. They identify a physically continuous, homogeneously managed area. However, multiple crops may be cultivated on individual *Plots* (Fig. 4); the area share of a single crop is reported in a separate variable and values below 0 and above 100 were replaced by NA. If a household grows more than one crop, it is possible that the total of individual crop area shares per *Plot* in percent exceeds 100 percent. This was not corrected or removed as an inconsistency as this might indicate that a household practises some form of multiple cropping, such as double or triple cropping.

## Data Records

The processed dataset and all scripts used to clean and compile the data are available at GitHub [https://github.com/koendvos/AfricanCropCalendar]. The data are also on Figshare [10.6084/m9.figshare.28659557].

### Dataset

[AfricanCropCalendar.csv]

https://github.com/koendvos/AfricanCropCalendar/

A table with 451,550 observations and 26 variables (Table 3). Instructions for downloading the raw data used to produce the dataset and the scripts for processing are provided in the repository subfolders, named for each country with ISO3 abbreviations (e.g. ETH, MWI).

**Table 3:** Column names and variable definitions.

Column Name	Definition
hhid	Unique household identifier
crop	Original crop names including varieties and local names
crop_simplified	Simplified crop name identifying crop types without further details
planting_month	Month of planting
planting_year	Year of planting
harvest_month_begin	Month in which the household began harvesting the crop
year_harvest_begin	Year in which the household began harvesting the crop
harvest_month_end	Month in which the household began harvesting the crop
harvest_year_end	Year in which the household began harvesting the crop
country	Country of residence
adm1-adm4	Name of the administrative units (such as state or region) of residence, up to four per country. Adm4 is the enumeration area (EA) from which the household was sampled.
lat	Latitude of the reference administrative unit (WGS 84)
lon	Longitude of the reference administrative unit
GPS_level	Reference point for GPS measurement, either plot or household level (aggregated to the enumeration area centroid), enumeration area, or other/unknown.
plot_area_reported_localUnit	Self-reported area in local units
localUnit_area	Name of local unit used to report area
plot_area_measured_ha	Area of plot as measured using a GPS unit in hectares
plot_area_reported_ha	The self-reported area, either direct respondent estimate or converted from local units
dataset_name	ID of the source dataset
dataset_doi	Digital Object Identifier of the dataset

### Metadata

[Metadata.xls]

https://github.com/koendvos/AfricanCropCalendar/

An Excel file with metadata: description, data type and units for each harmonized variable, source variable (label and description) from each survey used to generate harmonized variable, original questions asked in questionnaire for each source variable, percentage missing data for each harmonized variable.

### Lookup table for crop classification

[CropGroups_lookup.csv]

https://github.com/koendvos/AfricanCropCalendar/.

A table with 237 observations and 6 variables matching the crop names used in the dataset to the FAO’s Indicative Crop Classification (ICC) (see section “Crop names and classifications”):

- crop: original crop name in AfricanCropCalendar.csv
- group: Level 1 ICC
- group name: Level 4 ICC
- class_name: Level 2 ICC
- subclass_name: Level 3 ICC
- code: ICC 1.1 Code

### Lookup table for names of administrative units and geographic coordinates

[Coordinates_lookup.csv]

https://github.com/koendvos/AfricanCropCalendar/

A data frame with 1009 observations and 6 variables for matching the administrative unit names used in the dataset to official national names:

- id: row ID
- adm2: the original name of the administrative unit level 2 in AfricanCropCalendar.csv
- adm2.hdx: matched official name of the administrative unit level 2 in the HDX dataset
- lat.hdx: latitude of centre point of administrative unit level 2 in HDX dataset
- lon.hdx: longitude of centre point of administrative unit level 2 in HDX dataset
- country: country name

### Data Processing Subfolders

Code for creating the datasets are located in the subfolders

## Technical Validation

The following sections describe additional steps that were taken by the national statistics agencies to reduce measurement, processing and sampling errors during the data collection and by the authors to ensure consistent data entries in addition to the steps described above.

### Reducing measurement and processing errors

The survey design, process and implementation are detailed in technical documents provided for each survey separately in the World Bank´s microdata catalogue (https://microdata.worldbank.org/index.php/catalog/) where also the raw data can be downloaded. Technical documents include the enumerator manual that explains each section of the questionnaire, inclusion and exclusion criteria, and outlines field duties and a basic information document that explains the sample design, field work, data management and any problems and challenges faced during the data collection. Some steps for reducing measurement error are in particular relevant for the growing period dataset outlined here.

It is often difficult for farmers to estimate the size of the land they cultivate, and the size of individual agricultural fields of self-reported land and field size are not always reliable and can lead to measurement errors. The LSMS-ISA surveys were designed to minimise such errors by not only asking for the self-reported field size but also by measuring field sizes using hand-held GPS devices or, if a small field, by compass and rope.

### Planting and harvest dates

We have identified inconsistencies in planting and harvest dates based on expected ranges of normal results and our understanding of common error types. A common error was inconsistencies in months and years. We recoded all entries with numbers for planting and harvest months that were outside the expected range 1–12 to missing, with two exceptions. The 13^th^ month in Ethiopia, is the month Pagume and was recoded to September. In the Ugandan National Panel Survey 2015–2016 dataset harvest months larger than 12 indicate a harvest month in the following year, so we recoded them based on the respective planting month and year. For example, an entry with planting month 9, planting year 2015 and harvest month 13 indicates that harvest occurs in January 2016.

We recoded all entries with unclear year format for planting and harvest year as missing values, for example implausible single or double digits 8 or 20. This led to standardised planting and harvest date formats of “mm” for months and “yyyy” for years. We then recoded all entries with harvest dates later than the end of the survey as missing values. This predefined time window is based on the survey description and basic information documents provided for each survey. Planting years earlier than the beginning of the survey were left unchanged as they might indicate the establishment of an orchard or plantation for tree or orchard crops.

The harvest month and harvest year cannot be earlier than the planting month and planting year, they are otherwise both removed. Sometimes planting years in the 1980s or 1990s are given and should be validated for specific regions and crops. The earliest valid planting year was set to 1900. In Mali and Ethiopia, planting year, respectively the harvest year is missing. In Niger planting and harvest years are both missing. The most promising countries for an analysis of temporal trends in planting and harvest dates over a period of five to ten years are Malawi, Uganda and Nigeria.

### Comparison to existing national crop calendars

**Fig. 5:**
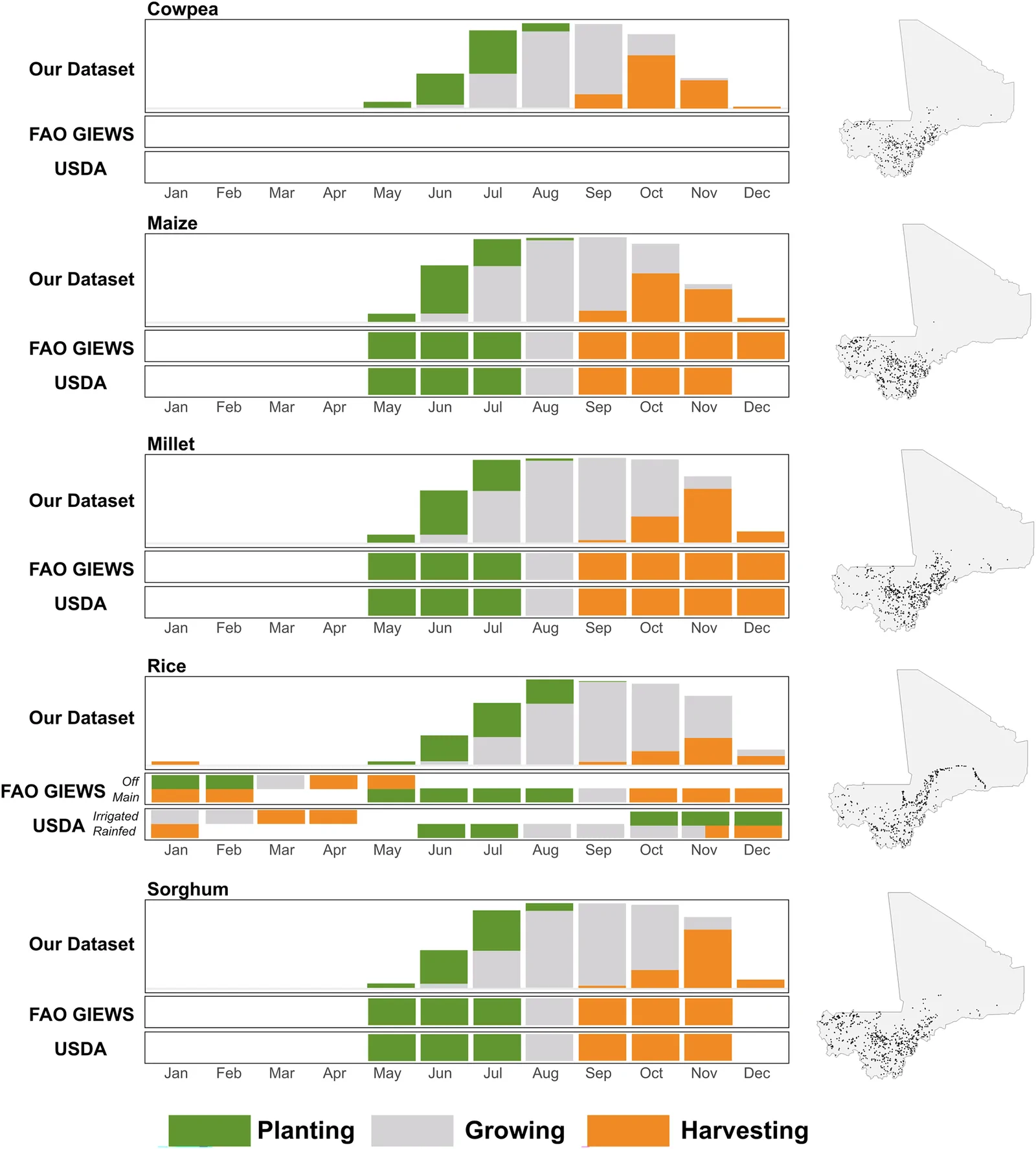
Cropping calendars for cowpea, maize, millet, rice, and sorghum in Mali based on this dataset^1^ - all waves (top row), compared to FAO GIEWS (middle row) and USDA (lower row) cropping calendars. Green indicates planting/sowing, grey indicates growing, and orange indicates harvesting. FAO GIEWS and USDA crop calendar indicate the month of planting, growing and harvesting. Our dataset indicates the share of crop area planted, growing or harvested in each month. Maps show the location of households cultivating each crop in our dataset. National estimates are not weighed to admin level representativeness in the data.

There are a range of datasets reporting planting and harvesting months on a national basis and we compared our dataset to the FAO GIEWS and the USDA crop calendars using the example of cowpea, maize, millet, sorghum and rice in Mali. In order to perform this comparison, we consider the data from the 2014 and 2017 waves together to exemplify the possibility of using our dataset to generate year agnostic crop calendars. The agreement between the cropping calendars is very high; the months identified as the planting months (in green) and harvest months (in orange) in the FAO GIEWS or USDA calendar are also the months with at least a portion of crop area planted or harvested in our dataset (Fig. 5). Our dataset has much more granularity by reporting planting, growing and harvesting periods by administrative units and enumerator areas and includes a larger variety of crops, including tree crops, horticulture crops, and various perennials, where existing cropping calendars have information mostly on cereal or oil crops.

There are some cases of disagreement between cropping calendars, and we will discuss them below as examples of uncertainty. These discrepancies arise because calendars were created using different data and information. By highlighting these cases, we aim to make data users aware of the need to carefully evaluate the characteristics of each data product to best correspond to their research interest and data needs. For planting of maize, millet and sorghum there is agreement for the main planting and harvesting seasons, but our dataset^1^ indicates an additional small portion of the crop area still not cultivated in August whereas the two other datasets indicate that planting has been finished by July. Another disagreement is the harvesting of sorghum which according to our dataset extends into December when the two other datasets indicate that harvesting finishes in November. Further, there is disagreement in the rice calendar in the first quarter of the year- corresponding to the growing season of irrigated rice (USDA) and off-season rice (FAO GIEWS). Planting of off-season rice is reported to occur in January and February in the FAO GIEWS but not in the USDA or our dataset. Harvesting of irrigated rice is reported to occur in March and April in the USDA calendar but not in the FAO GIEWS or our dataset. This particular disagreement can be traced back to the fact that the majority of parcels in the Mali surveys are registered as rainfed (90% for the 2014 wave, and 99% for the 2017 wave), but disagreement between USDA and FAO GIEWS also indicates that calendars are less clearly defined for off-season or irrigated rice.

## Usage Notes

### Mapping the data

The planting and harvest dates, crop types and average field sizes per household can be mapped using the geographic coordinates or the names of the smallest administrative units provided in the dataset, that is 4^th^ level administrative units in Uganda (parish), 3^rd^ level administrative units in Ethiopia (woreda), Mali (communes), Niger (communes), and Nigeria (local government areas) and 2^nd^ level administrative units in Malawi (districts).

We provide a lookup table to match these administrative unit names to official naming conventions in each country and provide the geographic coordinates of the centre points of each administrative unit to complement the ones provided already in the raw data. We have sourced official national administrative unit names from the Humanitarian Data Exchange website, v. 2.2.3 (HDX). We used approximate string matching as in the R package “stringdist” and a Jaro-Winkler distance of equal to or less than 0.1. This method will handle typos, alternate spellings and language variants such as between “east gojjam” and “east gojam”.

Additional geographic data for example on soil characteristics, climate, land use and land cover, socioeconomics and health can be linked to the dataset using the various geographic identifiers. For spatial analysis and queries, users should consider the offset range explained above and select the most appropriate spatial resolution of the analysis.

### Use cases

This data set can be used in agricultural research and development, climate and environmental studies, remote sensing and geospatial applications, socioeconomic and policy research and to some extent also in extension and adoption work. The most promising use cases are:

- Crop calendar optimization: analyse planting and harvest dates to refine regional crop calendars and improve seasonal planning.
- Yield estimation models: combine crop type, plot size, and growing season data with remote sensing, and weather data to model expected yields or use as input into process-based crop model, also to compare to existing rules for determining planting dates.
- Phenological studies: study crop growth cycles and their spatial-temporal trends and their drivers, also to detect land use changes.
- Crop system analysis: identify biodiversity-enhancing practices like intercropping and evaluating land use intensity and seasonal crop integration.
- Climate impact and adaptation analysis: assess how planting and harvest dates shift in response to changing climate conditions, and to what extent farmers have already adapted to changes in climate by adapting the planting dates, length of growing period, or crop type.
- Carbon footprint modelling: estimate emissions based on crop types, and timing of agricultural activities, as planting and harvest defines the crop’s active growth period influencing biomass accumulation, and timing of fertilizer, irrigation and pesticide application.
- Satellite calibration and validation: use as ground-truth data to validate remote sensing models for crop classification and phenology.
- Policy impact evaluation and extension targeting: assess the effectiveness of agricultural policies or subsidies by analyzing changes in cropping patterns, and identify farmers or regions with suboptimal planting practices for targeted outreach and training.
- Technology adoption studies: track how new practices or technologies influence planting and harvest dates and crop choices.

These studies can be part of food security, economic or ecological studies as the crops included in the dataset are cultivated for different purposes, as food for consumption by the household, as food for sale in local markets, as cash crops for sale in national and international markets, and as livestock feed. They include annual crops, perennial crops, tree and fruit crops, and fodder crops. We emphasise that the results of any analysis should be interpreted within the context of the dataset considering its bias and limitations described in this paper.

### Adding additional survey variables

Additional survey variables can be added to the dataset by extracting them via the unique household, field and plot identifiers. This provides the user with the opportunity to design the post-processing of other raw data and calculate the most relevant indicators for their research themselves.

## Data Availability

The code used to process the raw data and integrate into the final dataset is publicly available via https://github.com/koendvos/AfricanCropCalendar.

## Data availability

The dataset is available at GitHub [https://github.com/koendvos/AfricanCropCalendar] and Figshare [10.6084/m9.figshare.28659557]^1^.
